# Better living through ontologies at the Immune Epitope Database

**DOI:** 10.1093/database/bax014

**Published:** 2017-03-18

**Authors:** Randi Vita, James A. Overton, Alessandro Sette, Bjoern Peters

**Affiliations:** La Jolla Institute for Allergy & Immunology, Center for Infectious Disease, La Jolla, CA 92037, USA

## Abstract

The Immune Epitope Database (IEDB) project incorporates independently developed ontologies and controlled vocabularies into its curation and search interface. This simplifies curation practices, improves the user query experience and facilitates interoperability between the IEDB and other resources. While the use of independently developed ontologies has long been recommended as a best practice, there continues to be a significant number of projects that develop their own vocabularies instead, or that do not fully utilize the power of ontologies that they are using. We describe how we use ontologies in the IEDB, providing a concrete example of the benefits of ontologies in practice.

**Database URL:** www.iedb.org

## Introduction

The Immune Epitope Database (IEDB) project is a free online resource cataloging experimentally derived epitope information. The IEDB is sponsored by the National Institute of Allergy and Infectious Diseases (NIAID) (1) and is available at www.iedb.org. This resource hosts data derived from >18 000 scientific publications regarding the recognition of immune epitopes relevant to the fields of allergy, infectious diseases, autoimmune diseases and transplantation. An immune epitope is the portion of a pathogen, allergen or autoantigen that the immune system recognizes. When antibodies and T cells bind to epitopes immune responses are triggered that protect the host from infectious diseases or cancer but can also cause allergic and autoimmune diseases, or rejection of transplants. Understanding what epitopes the immune system recognizes facilitates a number of important research goals such as vaccine design, allergy immunotherapy and suitable donor–recipient pair selection in transplantation.

The IEDB has the challenge of describing diverse information on immune epitopes, the immunological context in which they were recognized, and the experiments used to characterize them with consistent and accurate language. To meet this challenge, we rely heavily on externally developed ontologies and resources. We use formal ontologies with logically defined terms, such as in the case of Ontology for Biomedical Investigations (OBI) (2) and Uberon (3), as well as resources with well-structured controlled vocabularies, such as the protein nomenclature provided by the reference proteome section in UniProt (4).

Ontologies provide standardized terminology for a specific domain, including textual definitions and a network of well-defined relationships between terms. The IEDB does not claim to be, nor does it aim to be, the expert on every facet needed to describe the >1 million experiments that we catalog. Rather, we turn to the expertise of different ontologies relevant to the database fields we use to represent our data. Each of these ontologies provides standardized nomenclature, definitions, synonyms and hierarchical relationships for database terms. Thus, we describe scientific concepts encountered in the literature consistently and accurately, using the proper terminology and with access to the synonyms researchers commonly use. Here, we will describe how using ontologies in the IEDB makes curation easier, enhances the user query experience and facilitates interoperability between our data and other databases or resources utilizing the same ontologies.

## Molecules

An immune epitope is the portion of a pathogen, allergen or autoantigen that the immune system recognizes. Antibodies typically bind to discontinuous residues of proteins, exposed on the surface of the protein in its native state. T cells recognize epitopes (typically peptides) presented by major histocompatibility (MHC) molecules on the surface of antigen presenting cells (APC). Antibodies and T cells also bind to non-peptidic molecules such as lipids and carbohydrates, as well as recognizing smaller compounds such as metal atoms and drugs in the presence of associated larger compounds. Thus, in order to describe epitopes, the IEDB must accurately describe the structure of the epitope itself as well as the source molecule of the epitope.

### Proteins

Peptidic epitopes, whether linear or discontinuous, are described as parts of proteins. Protein nomenclature is highly variable with different authors referring to the same protein using different names. For example, a commonly studied protein called Glutamate decarboxylase 2 by the UniProt reference proteome for humans is also referred to in publications as: ‘glutamic acid decarboxylase’, ‘65 kDa glutamic acid decarboxylase’, ‘Glutamate decarboxylase 65 kDa isoform’, ‘Glutamate decarboxylase-2 (pancreas)’, ‘GAD65 = autoantigen glutamic acid decarboxylase’, ‘Glutamate decarboxylase pancreatic islets and brain GAD’, ‘65 kDa, isoform CRA_c’, ‘GAD2 protein’, ‘GAD’, ‘GAD65’, ‘DCE2_HUMAN’ and ‘GAD-65’. We want curators to be able to type the name of the protein the author describes in the manuscript and have the database identify that the different names refer to the same entity.

**Figure 1.**
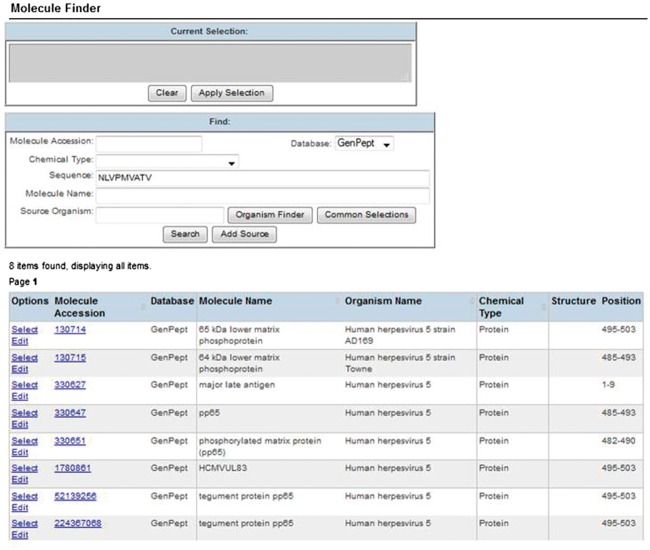
Curation molecule finder. Curators enter the epitope’s peptide sequence and click ‘Search’ to be shown only those proteins having 100% BLAST match to the epitope. This ensures accrate protein selection with minimal curation effort.

Certain species, such as viruses, have great genomic diversity, resulting in hundreds of different sequences for the same protein within the same species. For example, there are >300 *Influenza A virus* hemagglutinin protein sequences referred to in the IEDB in order to provide source sequences to all *Influenza A virus* epitopes studied. Fortunately, GenBank provides the necessary multitudes of deposited protein sequences that epitopes may be derived from (5). However, we cannot expect our curators or end users to browse >300 protein sequences to find the protein they need. To simplify this process for curators, we embed a Basic Local Alignment Search Tool (BLAST) (6) matching ‘molecule finder’ application within the curation interface, shown in Figure 1. This finder allows curators to enter their epitope sequence and click ‘Search’ to be shown only those proteins that are a 100% BLAST match to their input sequence. Thus, curators cannot select a mismatching protein. The molecule finder also has a number of other parameters that curators can use to narrow or filter the results by. This Finder ensures an accurate protein selection with minimal effort by the curator.

**Figure 2.**
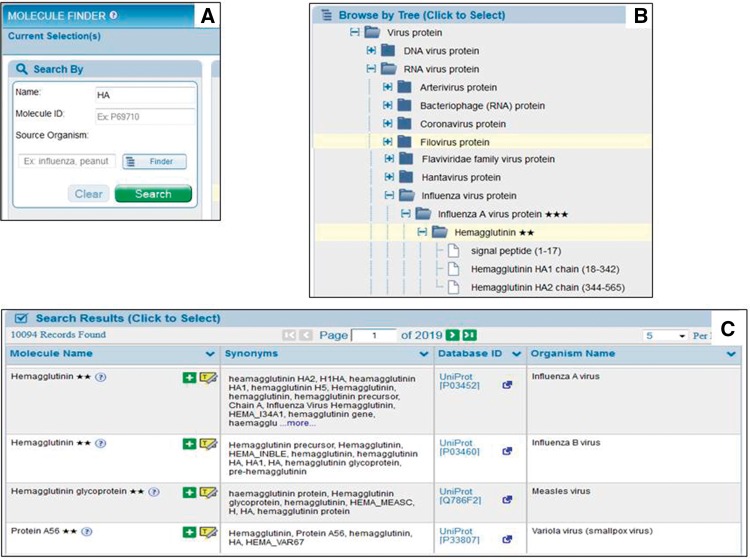
User molecule finder. Users enter the protein name and click ‘Search’ (**A**) or can browse the protein tree (**B**). The Search Results table in **C** shows all possible matches for what the user types.

For our end users, we additionally employ the benefits of the UniProt reference proteomes for each species present within the IEDB data. UniProt reference proteomes provide a framework to organize the set of proteins isoforms recorded from different species. This enables us to present an organism tree to the end users having each protein of that species’ proteome as a child node. As shown in Figure 2A, a user types the protein name and searches for it or, as shown in Figure 2B, they can browse the hierarchical protein tree. In order to allow our users to search by a wide variety of terms, we store all of the synonyms present in both GenBank and UniProt for all proteins having data in the IEDB as searchable term names. Thus, users can search by a variety of terms, such as ‘HA’ for ‘hemagglutinin’, allowing easy search and less typing. After clicking ‘Search’ users are returned the Search Results table, shown in Figure 2C. This table contains all hits for their search term and allows them to select a choice or to highlight it within the hierarchical protein tree.

Additionally, UniProt provides other annotations, such as functionally processed protein fragments, which we take advantage of to enhance the user query experience. Our curators assign a BLAST matching Genbank identifier to each epitope, for example GI:122875 for hemagglutinin. However, hemagglutinin is post-translationally processed into two fragments, HA1 and HA2 and while this GenBank record contains both fragments, the epitope is present in only HA1. Because UniProt stores these annotations for each protein, we are able to break proteins into each of their processed fragments in our search interface, adding new value not necessarily present in the original publication and without the curator having to take any action. Thus our users can search for all epitopes from hemagglutinin, HA1 or HA2 (Figure 2B). Furthermore, by linking our epitope data to both GenBank and UniProt identifiers, it becomes interoperable with the many other datasets and annotations related to these commonly used protein identifiers, including Gene Ontology (GO) terms (7).

Thus, the IEDB relies on the external expertise of GenBank and UniProt to describe proteins. Each of these resources provides stable identifiers, protein names, synonyms and sequences. We combine the merits of these resources to organize and represent for the >35 000 different proteins (89 272 different GenBank identifiers) utilized by IEDB data.

### Non-peptidic

**Figure 3.**
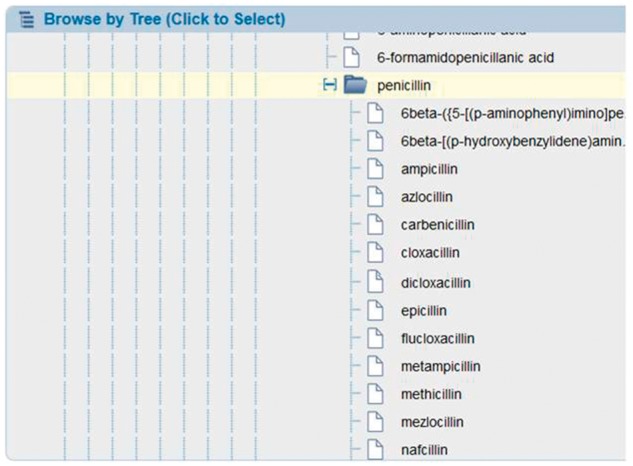
ChEBI molecule finder. Users can search on all penicillin drugs by selecting the higher node in the embedded chEBI hierarchy.

Peptidic epitopes are described using single letter abbreviations for amino acids, such as ‘A’ for alanine. This vocabulary is familiar to our team of curators, educated in the field of immunology and capturing peptide sequences in this code is accomplished routinely and accurately. But describing non-peptidic epitopes, which frequently have long and confusing terminology such as (1R)-1,5-anhydro-1-[(3S,4S,5R)-3-(hexacosanoylamino)-4,5-dihydroxynonadecyl]-d-galactitol, requires detailed knowledge in chemistry that our curators typically do not have. Therefore, we rely on the expertise of the chemists at Chemical Entities of Biological Interest (ChEBI) (8). Our team of immunologist curators needs only to know the correct ChEBI identifier for a non-peptidic epitope and by entering that identifier, they are provided with the proper nomenclature, synonyms and the chemical structure for that epitope. Through a productive and valuable collaboration with ChEBI, their curator provides our curators with correct identifiers on an ongoing basis. For each non-peptidic epitope, the ChEBI curator reads the manuscript and creates the entry within ChEBI or identifies the matching existing entry. Relying on the external expertise of ChEBI ensures accuracy in what our immunological curators enter into the IEDB. To benefit our end users, we embed the ChEBI ontology within the search interface, allowing users to browse the non-peptidic structures via the ChEBI structure hierarchy and utilize synonym searching. This allows users to find all epitope data on recognition of groups of structures, such as penicillin drugs, without having to search on each one separately (ampicillin, cloxacillin, etc.), as shown in Figure 3.

Non-peptidic epitopes are described in the IEDB utilizing >3000 ChEBI identifiers for structures such as penicillin, nickel, beryllium, cardiolipin and alpha-Galc. Many of these structures also play interesting roles in the real world, such as being drugs, dyes or antiseptics. These roles are already incorporated into the ChEBI ontology and we hope to integrate the ability to search by roles into the IEDB search interface next year. Additionally, as ChEBI identifiers link to resources, such as PubChem (9), Reactome (10), ChEMBL (11) and many others, our dataset automatically becomes linked to these many other resources.

## Organisms

Epitopes that are derived from proteins or natural non-peptidic structures, such as liposaccharide (LPS) have a natural organism as their source. Accurately describing the >3500 organisms IEDB epitopes are derived from, particularly their strains, is a daunting task. Therefore, we rely upon the expertise of the National Center for Biotechnology Information (NCBI) taxonomy (12). NCBI provides the standardized nomenclature and hierarchy needed to describe organisms properly. Strain names can be quite complicated, such as ‘*Influenza A virus (A/duck/Hokkaido/10/85(H3))*’, containing dashes, slashes and parentheses, and is not something we would want our curators attempting to type accurately. However, accurately capturing this data is critical to our users as the immune response to different strains varies and greatly affects vaccine design. We cannot expect the authors of publications nor our curators to consistently write strain names in such a format. To solve this problem, we embedded the NCBI taxonomy into our curation interface using a custom app that consumes the ontology and allows the user to select terms by navigating a tree view of the ontology, as well as allowing search by terms and synonyms. Queries are then executed over a SQL representation of the ontology in which parentage of terms is included, allowing query for all children of a selected term. This helps our curators find and select the correct terms, rather than requiring curators to write them out.

To enable our users to search on organisms within the IEDB, we embedded the NCBI taxonomy as an organism finder application into the search interface, showing only active nodes for which there exists curated data. While the NCBI taxonomy is correct and very helpful, because it is so extensive, we first found it to be cumbersome to use. Taxonomists may be familiar with all levels of classification, but most immunologists are not. Therefore, we generated an immunologist friendly hierarchy by pruning out nodes that were not directly used by IEDB data, providing common names for more unfriendly terms, and highlighting nodes with a great deal of immune reactivity data. We were able to show improved usability by testing users on the original NCBI taxonomy versus our pruned tree and found significant improvement in functionality, as previously described (1).

In an immune response, the host is the organism whose immune system recognized the epitope. These organisms are also described by NCBI taxonomy, with the exception of inbred laboratory strains like Balb/c mice, which are not present. Currently we extend the NCBI terminology utilizing internal IEDB identifiers, but we are in the process of mapping mouse strains to Mouse Genome Database Group (MGD) identifiers, whenever possible (13). As we are not experts on inbred mouse strains, we are looking forward to be able to utilize stable MGD IDs along with whatever additional metadata they can provide on each strain. MGD identifiers are being linked with information regarding human phenotypes and the correlating human diseases, thus resulting in tremendous value for our users. In the future, we hope to be able to do the same with rat strains and other laboratory species.

## Diseases

**Figure 4.**
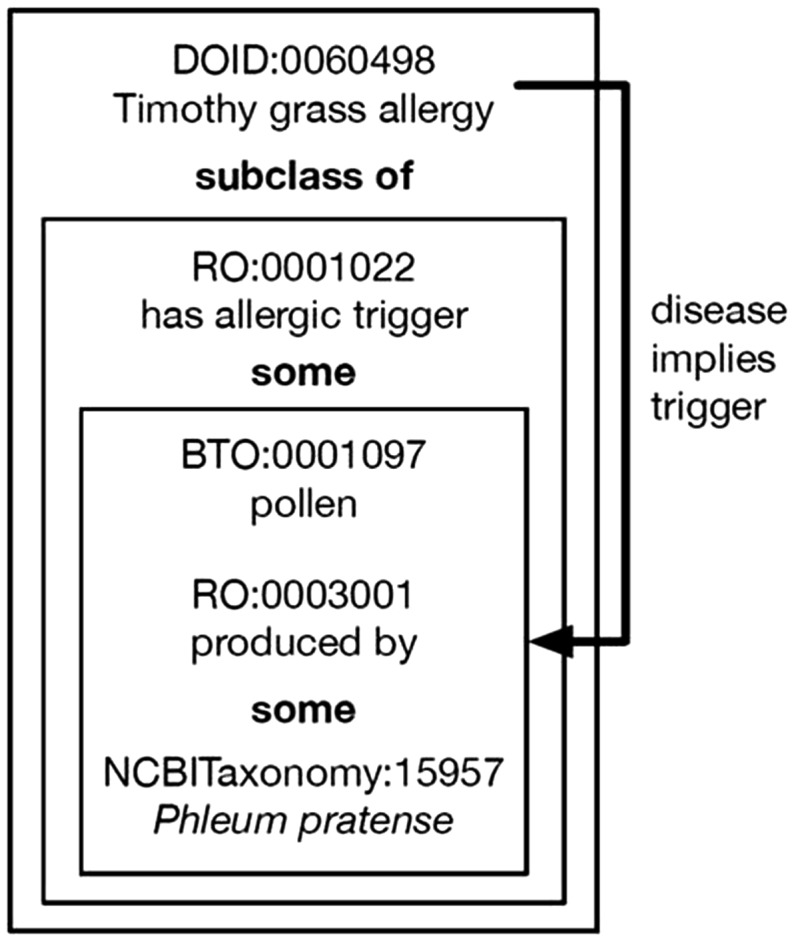
Logical definition of disease. Timothy grass allergy is defined as having pollen produced by *P. pratense* (Timothy grass) as its allergic trigger. This allows for inference of the trigger when curators select Timothy grass allergy and can also be used to generate a validation error if curators attempt to select a different allergic trigger for this disease.

The disease state of the host in an immunological experiment is integral toward understanding the data. For example, if a host had the disease malaria, antibodies toward *Plasmodium falciparum* epitopes, the causative agent of malaria, would be present, but in a host who did not have malaria, such antibodies would not be expected. In order to consistently and accurately describe disease states of hosts encountered in the literature, we depend upon the expertise of the Human Disease Ontology (DO) (14). As with other ontologies, we gain the proper nomenclature, synonyms, textual definitions and a searchable hierarchy. But, by also logically defining diseases as being triggered or caused by certain agents, additional features become possible. Allergic diseases, for example, are triggered by specific allergens. Using the Web Ontology Language version 2 (OWL2) (15), Timothy grass allergy (DOID:0060498) is logically defined a subclass of ‘“has allergic trigger” some (pollen “produced by” some “*Phleum pratense*”)’, where the relations ‘has allergic trigger’ (RO:0001022) and ‘produced by’ (RO:0003001) come from the Relations Ontology (16), the class ‘pollen’ (BTO:0001097) comes from the BRENDA tissue/enzyme source (17), and the class ‘*P. pratense*’ (NCBITaxonomy:15957) comes from the NCBI Taxonomy (12), as shown in Figure 4. From that logical definition we know that our curators should only enter this disease as being triggered by that allergen. This logic allows us to find and correct errors and develop curation rules that prevent future errors. Additionally, we can create inference rules that allow curators to enter minimal information, such as the host has Timothy grass allergy, and then auto-populate the trigger as being pollen from Timothy grass, decreasing the number of fields curators need to enter.

For our end users, the DO hierarchy provides the ability to search on groups of diseases based upon expected terminology and common practice, such as autoimmune disorders or viral infections. Additionally, searching by a large number of synonyms, including ICD-10 codes, is enabled.

## Geolocation

The geographic location of human hosts is of interest to IEDB users as certain diseases are more prevalent in certain areas and/or may present specific challenges based upon location. Therefore we embed the Gazetteer (Gaz) ontology into our curation and search interfaces (18). Similar to the NCBI taxonomy, the entirety of Gaz contains a very large number of nodes, so we found it necessary to limit the number of nodes we access to geographic regions, continents, and countries. This provides the level of detail that our data need, while providing curators and users with a manageable finder interface.

## Assay

**Figure 5.**
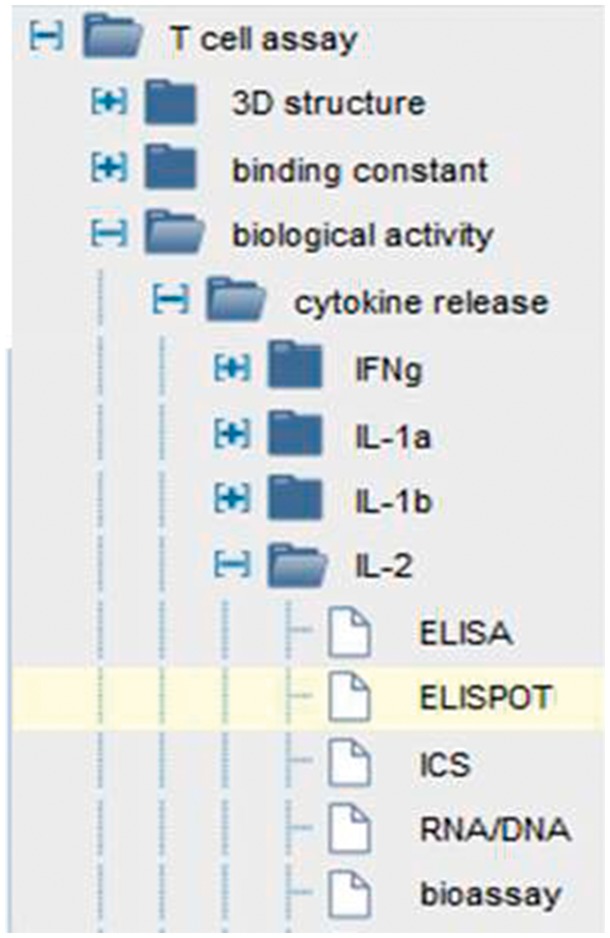
OBI driven assay finder. Users can search on all T cell assays, all cytokine assays or selectively on IL-2 assays.

The IEDB is a database of immune epitope experiments and currently houses over one million experimental assays. In order to consistently and accurately describe all of the assay types that we encounter in the literature, we utilize the OBI (2). OBI provides a logical hierarchy, synonyms, textual and logical definitions, and examples for all of the assay types needed by the IEDB data. We embed this ontology into the curation and search interfaces as an assay finder application that simplifies both curation and searching. As shown in Figure 5, users can search our data by a variety of hierarchical levels of logical grouping of assays, such as all T cell assays, or all assays that measure a certain response type, such as cytokine release, or types that measure a certain cytokine (e.g. IL-2). As new assay types are developed, we contribute new assays to OBI on an ongoing basis. To date, we have contributed >400 assay types to OBI, demonstrating how practical usage of formal ontologies by scientific resources is beneficial for both the resource and the ontology (19). Again, as more resources continue to also utilize OBI, our datasets can be linked via these formal identifiers.

## Cells

In order to describe T cell responses to epitopes, we must consistently portray both the type of cell studied and the tissue from where it was isolated. It is not uncommon for the T cell response to be strong in one tissue and weak in another, so these facets are important to our users. Thus, we have mapped all tissues encountered in the literature to Uberon (3), which allows for different species to utilize the same term for the homologous structure. Additionally, we mapped all cell terms used by IEDB data to either Cell Ontology (20) or Cell Line Ontology (21) terms, depending on if the cell was studied direct *ex vivo* or was a stable cell line. We found it promising that all tissues and nearly all cells encountered in the over 1 million experiments we house were easily found in these ontologies. Currently, this information is not displayed within the curation or search interfaces, but we hope to embed a Cell Finder during the next year.

## MHC

T cell epitopes are recognized in the context of MHC, present on the surface of APC. The IEDB describes the MHC restriction of its T cell data, as well as capturing experiments demonstrating epitope binding to MHC molecules and APC processing and presentation of an epitope within a specific MHC molecule. MHC nomenclature is complex, varies greatly by species, and is often confusing. Thus, rather than relying on authors or curators to accurately describe these complex terms, such as HLA-DQA1*05:01/DQB1*02:01, we devised a new ontology called the MHC Restriction Ontology (MRO) (22). This ontology models MHC protein complexes as two protein chains encoded by different loci, that come together to form the complete MHC molecule. This data is presented in a hierarchical tree and allows for synonym searching. As with other ontologies, this allows our curators to find and select the correct terms, rather than needing to type such complex terminology. Additionally, the MRO tree serves as an educational device for our end users, not only to communicate what data we have in the IEDB, but also to communicate the proper nomenclature for MHC molecules, which is still evolving.

## Discussion

As more and more resources represent biological and chemical information utilizing formal ontologies, interoperability between data sources is facilitated and new conclusions can be made about the data contained in independent resources. For example, the IEDB contains data stating that benzylpenicillin is an epitope recognized in the context of an allergic reaction. Because we utilize ChEBI ontology and annotations, the fact that benzylpenicillin is a drug, an antibacterial drug, a beta-lactam antibiotic, etc. are gained knowledge, as far as the IEDB data are concerned. The fact that benzylpenicillin is an epitope and an allergen is gained knowledge for ChEBI.

Using the same, standard ontology terms across datasets are a crucial step toward the vision of Linked Data (http://linkeddata.org), enabled by technologies such as the Resource Description Framework (RDF) and RDF databases (‘triple stores’). These techniques and tools make it possible to bring together heterogeneous datasets, query their combined data and make new discoveries. This represents the ultimate goal of all publically available scientific resources, to further knowledge. We hope to capitalize on this capability and are currently working to join the IEDB data with other overlapping projects, such as PlasmoDB (23) and ImmPort (24) to further the value of each project.

Thus, by utilizing formal ontologies, we have simplified the curation process and ensured improved data accuracy, while simultaneously adding value to our datasets, enabled database interoperability and made our search interfaces more user friendly.

## Funding

National Institutes of Health (HHSN272201200010C).

*Conflict of interest*. None declared.
